# Implementation of Motivational Interviewing Training in an Undergraduate Nursing Curriculum: Identifying Adolescents at Risk for Substance Use

**DOI:** 10.3390/ijerph15081623

**Published:** 2018-08-01

**Authors:** Denise Seigart, Max Veltman, Janet Willhaus, Colene Letterle

**Affiliations:** 1 College of Health Sciences, East Stroudsburg University, East Stroudsburg, PA 18301, USA; 2 School of Nursing, Boise State University, Boise, ID 83725, USA; maximilianveltman@boisestate.edu (M.V.); janetwillhaus@boisestate.edu (J.W.); 3 West Ada School District, Meridian, ID 83642, USA; Letterle.Colene@westada.org

**Keywords:** motivational interviewing, SBIRT, nursing curriculum, substance use prevention

## Abstract

Motivational interviewing (MI) has been increasingly utilized by health care practitioners for many years. MI has been practiced by social workers, nurses, physicians, psychologists, substance use counselors, and many other health care practitioners. Unfortunately, many health care practitioners do not have adequate training in motivational interviewing, and therefore feel ill equipped to utilize this approach when faced with clients who are in need of assessment and coaching. This paper discusses our experiences with a pilot project to implement MI training within an Adolescent SBIRT (Screening, Brief Intervention, Referral to Treatment) content addition to the undergraduate nursing curriculum. It includes discussion of the evaluation, which measured student attitudes towards substance users with the Substance Use Attitudinal Survey (SAAS), student satisfaction with the newly implemented curriculum, and implications for sustainable inclusion of this content and simulation experiences at the undergraduate level to promote MI use by future health care practitioners. Pre- and post-tests (SAAS) were conducted with 51 nursing students, and 56 students completed the satisfaction survey. Overall, students were very satisfied with the implementation of the curriculum, however, we did not see significant changes in SAAS test scores. This may, however, be a positive indicator of a balanced attitude toward substance users. Continuing evaluation of the curriculum change is needed.

## 1. Introduction

Motivational interviewing (MI) has been increasingly utilized by health care practitioners for many years. Originally developed by Miller [1], motivational interviewing has been practiced by social workers, nurses, physicians, psychologists, substance use counselors, and many other health care practitioners. The definition of motivational interviewing has evolved and changed since the original work on its usefulness as an approach to behavior change. Through experience and research, the fundamental principles and methodologies of MI have been applied and tested in a variety of settings and research findings have demonstrated its positive effects. MI is now recognized as an evidence-based practice in the treatment of individuals with substance use problems, and is defined as follows:

Motivational interviewing is a collaborative style of communication with particular attention to the language of change. It is designed to enhance personal motivation for and commitment to a specific goal by exploring the youth’s own reasons for change within an atmosphere of caring and compassion.

[2]

Unfortunately, many health care practitioners do not have adequate training in motivational interviewing, and therefore feel ill equipped to utilize this approach when faced with clients who are in need of assessment and coaching.

This paper discusses our experiences with a pilot project to implement MI training and the Adolescent SBIRT (Screening, Brief Intervention, Referral to Treatment) [3,4] model within the undergraduate nursing curriculum at Boise State University. After receiving a grant from NORC at the University of Chicago, faculty at Boise State agreed to incorporate MI and Adolescent SBIRT training into the undergraduate baccalaureate nursing curriculum. We include discussion of the evaluation of this curriculum change, which measured student attitudes towards substance users as measured by the Substance Use Attitudinal Survey (SAAS) [5] and administered locally developed student satisfaction surveys. We also discuss implications for sustainable inclusion of this content and simulation experiences at the undergraduate level to promote MI use by future health care practitioners. By adding MI and SBIRT content to the nursing curriculum, this pilot project sought to increase nursing students’ knowledge and skills related to MI and SBIRT in adolescent and young adult patients. Students received lectures on MI and SBIRT content and practices, role play experiences, lab simulations, and clinical practice opportunities during their community health rotation. This project was approved by the Boise State Internal Review Board process.

## 2. Review of the Literature

Researchers note that practitioners who do not receive adequate training and experience with MI are ill equipped to use the method and less effective in their work with those at risk of substance use, but those who have training and field experience opportunities with faculty feedback demonstrate enhanced skills [6,7]. Motivational interviewing has been shown to be a viable method of approaching teens with the aim of behavior change and reducing or eliminating substance use [8,9,10,11,12].

One study that examined the effect of brief motivational interviewing on high-risk teens in a primary care clinic found that teens involved in the project “reported less marijuana use, lower perceived prevalence of marijuana use, fewer friends who used marijuana, and lower intentions to use marijuana in the next 6 months, as compared to teens assigned to usual care.” [12] (p. 61). Other studies have also demonstrated an increased ability to dissuade teens from substance use by using motivational interviewing. For example, in a study that randomly assigned forty adolescent patients to receive either brief advice or a motivational interview, the authors report “brief smoking interventions with teen patients was supported by high rates of recruitment, retention, and quit attempts, and long periods of continuous abstinence.” [8] (p. 574).

Similar to national statistics, in the state of Idaho, where this project was conducted, the 2017 Idaho Youth Risk Behavior Survey reported that 18.5 per cent of teens reported using some form of tobacco or vapor in the past 30 days, 26.5 percent reported alcohol use in the past 30 days, and one in five (22%) Idaho high school students were offered, sold, or given an illegal drug on school property during the previous 30 days [13]. School nurses in Idaho typically utilize whatever communication tools and training they have in their “tool box” from their undergraduate education and nursing experience. This does not often include training in MI. Some districts have the support of state funded drug and alcohol prevention counselors at the secondary schools, thus, nurses often partner with the counseling staff to assess student needs and access services, but for brief assessment and referral school nurses and counselors have not been routinely trained to use MI. As noted by D’Amico et al., insufficient training is a common problem among health care providers:

It is important to improve the capability of primary care providers and associated health care staff to identify youth at risk for alcohol and other drug (AOD) use because use during adolescence is associated with academic problems, poorer mental health, future use of other illicit drugs (including heroin and cocaine), and a higher likelihood of use or dependence in adulthood. Unfortunately, most adolescents are not screened for AOD use in primary care settings, and significant numbers of at-risk youth remain unidentified and never receive appropriate preventive or treatment services. Lack of screening and preventive services is even more profound among younger adolescents age 11 to 14 and socioeconomically disadvantaged youth. Lack of primary care screening typically is caused by provider time constraints, discomfort discussing AOD use, insufficient training, or lack of referral options.

[14]

## 3. Implementation of the Boise State Program

As mentioned previously, at Boise State University, we were presented with the opportunity to implement SBIRT training with a grant awarded by NORC (University of Chicago, Chicago, IL, USA). In response to receiving this grant, we agreed to design and deliver lecture content, simulations, and clinical field experiences for nursing students over a three-semester period of time. The Boise State undergraduate baccalaureate nursing program is composed of both new high school graduates and adult learners changing careers. Approximately 310 students are enrolled in the program at any given time (fourth through eighth semesters), and these range in age from 18–50. Approximately 20% of the student population is male in any given semester, and the large majority of students are white, although there are representatives of Hispanic, Latino, Native American, Asian, and African-American populations each semester (approximately 15% total).

During the semesters held January of 2016 thru September of 2017, Boise State faculty integrated MI and SBIRT content into several courses via Project MINE (Motivational Interviewing in Nursing Education) including behavioral/mental health (didactic and simulation labs) and community health (clinical experiences). Students initially received training and role play experiences with MI and the Adolescent SBIRT model during their behavioral/mental health didactic course, with additional exposure during corresponding lab simulations. Additional practice opportunities were later provided in their community health clinical experiences (with school health as the emphasis). Students also had the opportunity to implement their new understanding of MI in their medical-surgical clinical labs, if adolescent or young adult patients presented themselves in the medical-surgical settings.

**Figure 1:**
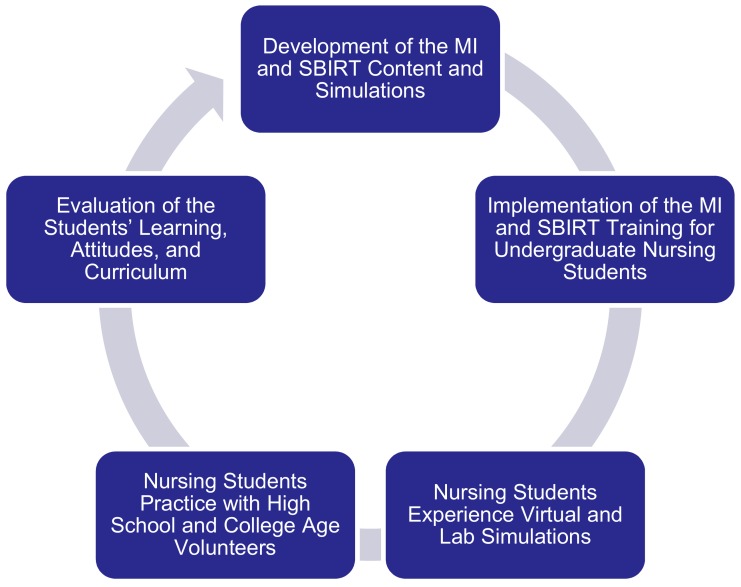
Process for implementation of the motivational interviewing (MI) and SBIRT (Screening, Brief Intervention, Referral to Treatment) training.

The overarching goal of Project MINE was to increase nursing students’ knowledge, understanding, and skill related to addiction screening, referral, and treatment in adolescent patients utilizing MI and the Adolescent SBIRT model [4] and included the following objectives:

1. Faculty would integrate use of MI and the Adolescent SBIRT model throughout the Boise State undergraduate nursing curriculum during the spring and fall semesters from January 2016 through September 2017.
2. Faculty would identify opportunities for students to practice MI and the Adolescent SBIRT model with adolescent patients during existing clinical experiences and/or simulations (see Figure 1 below).
3. Students would demonstrate improved knowledge and skills in utilizing MI and the Adolescent SBIRT model as well as changes in their attitude towards substance users as evidenced by changes in pre- and post-test scores on the SAAS (Substance Use Attitude Survey).

As mentioned previously, nursing students were first introduced to the motivational interviewing process and how to use the CRAFFT screening tool for assessing early risk factors for substance use in their behavioral/mental health course. Originally developed by Knight et al. [15,16], CRAFFT is an acronym of the first letters of key words (car, relax, alone, forget, friends, trouble) in six questions developed to screen adolescents for high risk behaviors regarding substance use. Prior to learning how to use the CRAFFT tool, students learned about motivational interviewing methods, which include a caring and compassionate approach to youth without judgement regarding the use of substances. Nursing students are taught not to try to convince the youth to stop using substances, but rather to help them examine how much they are using, why they are using, and how much motivation there might be to change their behavior slightly [4].

Following the introduction of motivational interviewing and SBIRT methods during the lecture, students were provided with the opportunity to practice these skills during their lab experience for behavioral/mental health (a separate class). A virtual simulation was utilized for initial practice, and then experiences with standardized patients were provided for all students in order to reinforce their learning and enhance development of these skills. An online module (Kognito™) [17] allowed the learners to participate in three different SBIRT and motivational interviews designed to build knowledge and skills in motivational interviewing. Learners received a score for the final interview and were asked to repeat the module until a score of at least 75% was achieved.

An additional practical experience included three MI/SBIRT simulations with live actors. The simulation scenarios were authored by an expert in simulation pedagogy and piloted and reviewed by content experts prior to use. In the scenarios, the learners played the roles of student nurses at a campus health fair. Each learner had the opportunity to interact with three different live actors who had been trained to portray roles of new college freshmen. One was a young woman who had initiated drinking at college parties in the past few weeks. The second was a young person who smoked marijuana daily due to the stresses of school, and the third was a college student who was feeling a lot of peer pressure to start using substances. After each scenario, the live actor provided feedback to the learners on their use of MI communication techniques and the SBIRT model. After all learners had completed the practice sessions with the live actors, group debriefings were facilitated by their instructors in small groups of up to 10 students.

Following their exposure to MI and SBIRT methods in their behavioral/mental health course, student learning was reinforced with additional exposure to MI in their Care Coordination course. Finally, students were provided with a community field experience for their final practice opportunity during their community health rotation. The following discussion describes this field experience.

## 4. Implementation of the Community Health Clinical Project

In the spring of 2017, Boise State nursing students were able to practice their newly learned MI skills with members of the local community. These interviews were coordinated and scheduled as a student-driven health promotion project within the community health clinical course. In other words, a group of students took on the task of organizing the screening as a partial requirement for meeting some community health clinical course objectives. The adolescent participants who worked with the nursing students were either youths located at a single local high school or from one of two residence halls located on the Boise State campus.

The ability of nursing students to engage in motivational interviews with actual high school students was largely due to the long-standing relationship between the Boise State University College of Health Sciences and a nearby rural school district that has served as a clinical partner for many health promotion projects over the previous years. This district is located near the western border of Idaho and has between 810–830 K–12 students attending class each year. This relationship has evolved and continues to grow because of the college’s commitment to working with local community members in improving health and health care delivery. Over the years, the School of Nursing, the School of Social Work, as well as the Kinesiology department have had both undergraduate and graduate students engage in multiple service learning type of activities involving health promotion and health education in this district. Of particular note is the fact that the district has no school nurse, so the nursing students provide some of the only exposure the K–12 students have to nurses during their time together.

Boise State University has several on-campus residential housing units where residents are placed based on their field of study (living-learning communities). Each of these housing units has faculty advisors, many of whom live in the residence along with their students in an effort to enhance the learning activities and increase engagement with the student residents. Two of these faculty advisors agreed to encourage their resident students to participate in MI interviews conducted by nursing students.

## 5. High School Students

Following a comprehensive needs assessment of current health issues within the local high school, several undergraduate nursing students, in collaboration with key school administrators and faculty at the high school, developed a plan that would allow high school students to engage in private one-on-one interviews with Boise State nursing students. After acquiring needed approvals from administrators and faculty at the high school, consent forms were sent out with all eligible students at the high school for parental permission to participate. Eligibility depended mainly on if the high school student was in one of several classes in which the high school faculty agreed to participate. High school students were free to decline participation at any time. Additionally, a safety plan was developed for the nursing students to follow should any nursing student feel like an immediate intervention might be important based on their discussion with the adolescent participant. Over the course of the project, participating high school students would meet with nursing students in private rooms on the high school campus. These interviews would last between 5 to 20 min. Nursing students utilized the CRAFFT questionnaire while conducting the interviews with integration of the SBIRT readiness ruler as described by “NORC [4]” at the beginning of each interview.

Twenty-seven high school students volunteered to participate in the SBIRT interviews over the course of the project. No demographic information was obtained regarding each participant as part of the effort to encourage participation and maintain confidentiality. During the course of the interview sessions, one high school student was immediately referred for additional counseling services related to experiences the student disclosed during the MI interview. The nursing student involved utilized the protocol established prior to the implementation of the interviews by contacting the appropriate school counselor who had the ability and the resources to make important immediate follow-up referral(s) to assist the high school student.

## 6. University Students

In an effort to increase the number of nursing students who were afforded the opportunity to practice with youths or young adults in this MI curriculum integration project, 18-year-old college freshmen were offered the opportunity to be participants in the project as well. The process of coordinating and scheduling the interviews was similar to the efforts described for the high school student interviews. The faculty supervisors of two residence halls were approached by nursing faculty to participate in the MI project. After two separate presentations by the nursing students involved in the project to the students in the residence halls, the interviews were scheduled with volunteers. Over the course of a semester, nursing students would meet privately with the residents and follow a similar motivational interview process as detailed previously with the high school students. Twenty-seven (27) student residents participated in this activity. All residents were given the opportunity to opt out of the exercise if they desired. These two clinical opportunities provided the majority of the nursing students in the community health clinical lab the chance to practice MI in the “real” world with youths/young adults in the area. By the end of this semester, 25 nursing students out of 60 in the community health course took advantage of the opportunity to practice their motivational interviewing skills in these settings.

## 7. Survey Methods/Results

In order to measure any change in attitudes of nursing students towards those at risk of substance use, undergraduate nursing student participants in this pilot project were surveyed using the SAAS instrument [5]. This instrument is a 50-question survey with five subscales that was designed to measure attitudes among health professionals regarding treatment of patients engaged in alcohol and other drug misuse. Subscales include permissiveness or tolerance in accepting substance use, non-stereotyping, treatment intervention, treatment optimism, and non-moralism. The SAAS was administered prior to the teaching modules on MI and SBIRT in their first semester of exposure to SBIRT methods, and then again following their last exposure to MI and SBIRT methods during their community health clinical rotation. To date 51 participants from the undergraduate program have completed the SAAS. The SAAS pre- and post-test scores were analyzed using paired *t*-tests. There were no statistically significant differences in subscale scores for the undergraduate nursing participants.

In addition, all nursing students were asked to complete a locally developed satisfaction survey regarding their learning experiences and the simulations they completed during their behavioral/mental health course lab (56 students completed this survey voluntarily). This survey was developed to assess students’ satisfaction with the MI and SBIRT content as well as the simulations and other lab activities developed to reinforce the lecture content. Results of the satisfaction survey indicated that students were very satisfied with the content and simulation experiences. Specific scores are found below in Table 1.

Comments from students on the satisfaction survey.

- What students liked best:
  - Opportunity to practice with real people
  - Opportunity to practice with standardized patients first
  - Useful communication technique (MI)
- What students would change:
  - Would like more opportunities to practice with standardized patients
  - More opportunity to practice MI with “real” people

## 8. Discussion

Overall, this pilot study of the implementation of a nursing curriculum that includes MI training has been found to be valued by students, faculty, and clinical partners. Faculty have stated that students demonstrated a high degree of retention of the course content and skills, and students were noted by faculty to be utilizing the skills even outside of the required simulations and field experiences. One faculty member stated that a student reported she had been promoted at work due to her use of MI and SBIRT skills while she was functioning as a nurses’ aide. Students reported a high level of satisfaction with the course content, simulations, and field experiences and this satisfaction was also noted via end of semester course surveys. While student scores on the SAAS pre- and post-tests did not exhibit a statistically significant change, it may be important to note that their attitudes towards substance users did not become increasingly negative, which has been noted in other studies [18].

While the implementation of this project, as with any new endeavor, presented some challenges, the faculty did express a desire to continue the implementation of the MI training and SBIRT theory and clinical experiences after the grant period expired. One of the biggest barriers to the implementation of this project involved arranging the many interview experiences for youths in the local high school. It is expected that the faculty will downscale this project somewhat, due to the overwhelming workload created for the faculty member when this type of clinical experience is planned. Arranging the interviews of first year college residents was less cumbersome and will likely be continued. In addition, the virtual experiences provided via the Kognito virtual simulation are no longer available to nursing students since the grant has expired. The cost of purchasing the simulations is prohibitive, thus rather than continue with Kognito, the nursing faculty have planned alternative simulation experiences, which they have developed themselves.

## 9. Conclusions

Based on our experience with the implementation of this curriculum and based on the substance use crisis we are currently experiencing in the U.S., we feel it is important that all nursing programs consider implementation of MI and SBIRT content. Students’ survey answers indicated that the MI and SBIRT content and practice of skills benefitted students and was well received. Anecdotal accounts indicated that the skills students learned regarding MI were able to be utilized in their other clinical rotations, and even in their nursing aide positions during off hours. In addition, students reported that the MI skills were useful for motivating clients to change nutritional habits, exercise habits, and a host of other behaviors, which is consistent with other studies mentioned previously [8,9,10,11,12]. Students did demonstrate retention of information regarding MI and SBIRT and expressed satisfaction with the content and practical experiences. This pilot study seems to indicate that preparing more health care practitioners with MI and SBIRT skills can increase health care providers’ abilities to identify and assist those at high risk for substance use, but additional studies are needed regarding the lasting attitude change of healthcare providers in order to ensure that the use of MI and SBIRT methods endures. Ongoing evaluation efforts for this project will continue to document the implementation of this curriculum change and the results of these efforts for undergraduate nursing students. Faculty will continue to utilize satisfaction surveys and course evaluation surveys and are considering a different knowledge survey (as opposed to the SAAS) for future evaluation efforts.

## Figures and Tables

**Table 1:** Survey/Likert type 1–5 (with 5 being best) administered to students regarding satisfaction with the content and learning activities. *N* = 56.

**Virtual Sim Satisfaction**	
Identification of Substance Abuse	3.54
Teaching Conversation Tactics	3.95
Realistic Conversations	3.69
**Standardized Patient Satisfaction**	
Quality of Simulations	4.03
Satisfaction with Experience	4.05
**Satisfaction Results**	
Met Purpose	4.07
Enhanced Skills	4.20
Relevant	4.25
Better Practitioner	4.27
Will Use Methods	4.27
Useful for Future	4.25
Will Benefit Patients	4.31
Was Able to Implement	4.11
